# Mild TBI Changes Brain and Plasma Neurosteroid Levels in Mice

**DOI:** 10.1089/neur.2024.0151

**Published:** 2025-01-20

**Authors:** Kosisochukwu E. Umeasalugo, Igor Khalin, Burcu Seker, Philippe Liere, Antoine Pianos, Maria Sanchez-Garcia, Michael Schumacher, Inga Katharina Koerte, Nikolaus Plesnila

**Affiliations:** ^1^ Institute for Stroke and Dementia Research (ISD), LMU University Hospital, LMU Munich, Munich, Germany.; ^2^ cBRAIN, Department of Child and Adolescent Psychiatry, Psychosomatics and Psychotherapy, LMU University Hospital, LMU Munich, Munich, Germany.; ^3^ Graduate School of Systemic Neurosciences (GSN), LMU Munich, Munich, Germany.; ^4^ Munich Cluster for Systems Neurology (Synergy), Munich, Germany.; ^5^ U1195 INSERM and University Paris Saclay, Le Kremlin Bicetre, France.; ^6^ Psychiatry Neuroimaging Laboratory, Mass General Brigham, Harvard Medical School, Boston, Massachusetts, USA.; ^7^ German Center for Child and Adolescent Health (DZKJ), Partner site Munich, Munich, Germany.

**Keywords:** biomarkers, concussion, experimental, mild, mouse, neurosteroids, traumatic brain injury

## Abstract

Mild traumatic brain injury (mTBI) accounts for 80% of all TBI, may be associated with chronic impairments, and is difficult to diagnose due to a lack of objective markers. In this study, we investigated whether neurosteroids can serve as blood biomarkers for mTBI. Two cohorts of C57BL/6 mice were subjected to a model of mTBI combining impact with rotational acceleration or sham surgery. The first cohort underwent neurological testing for anxiety, balance, and locomotion before and after mTBI. For the second cohort, brains and plasma were collected 6 or 24 h after mTBI to measure steroid and neurosteroid levels by gas chromatography-tandem mass spectrometry. Traumatized mice exhibited significantly prolonged wake-up time from anesthesia, transiently increased beam-walk time, and mild astrogliosis compared with their control counterparts, but did not suffer from skull fractures, intracranial hemorrhage, or mortality. Isopregnanolone and 3β,5α-tetrahydrodeoxycorticosterone (ISODOC) were significantly decreased by more than 50% in brain parenchyma at 6 and 24 h after mTBI, while ISODOC was also significantly decreased in plasma (−75%). Therefore, ISODOC may be a candidate diagnostic biomarker for mTBI.

## Introduction

Traumatic brain injury (TBI) is a major cause of death and disability worldwide. Out of the 27–69 million TBI cases per year^1,2^ about 80% are categorized as mild TBI (mTBI).^3,4^ mTBI results from falls, collisions, sports, or road traffic accidents and may involve a brief loss of consciousness, headaches, dizziness, nausea, confusion, and restlessness.^5,6^ Most symptoms resolve within a few days to weeks after mTBI, but in 50% of patients these symptoms may persist beyond 12 months.^7–10^ In pediatric mTBI, children and adolescents have a higher risk of developing cognitive and behavioral symptoms^11,12^ which may continue up to 3 years following injury.^13^ Current tools for mTBI diagnosis such as the Glasgow Coma Scale or Pediatric Trauma Score have been laden with problems of interobserver inconsistencies and failure to accurately triage patients,^14,15^ thus the field of neurotrauma is in need of objective diagnostic biomarkers.

**FIG. 1.**
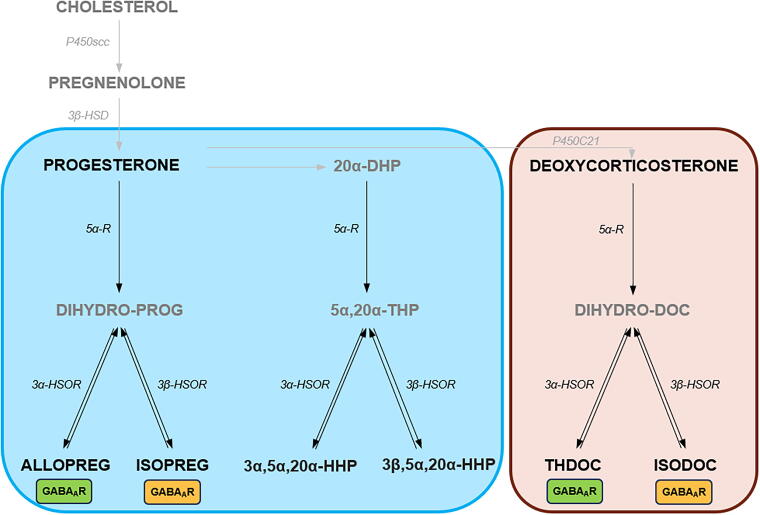
Schematic overview of neurosteroids. Neurosteroids include progesterone and deoxycorticosterone (DOC), and their metabolites. The corresponding enzymes are also shown. Neurosteroids acting on the GABA_A_ receptors are indicated by boxes (green for positive modulators and yellow for negative modulators). 5α-R, 5-alpha reductase; 3β-HSD, 3β-hydroxysteroid dehydrogenase; 3β-HSOR; 3β-hydroxysteroid oxidoreductase; 20α-DHP, 20α-dihydroprogesterone; 3α5α20α-HHP, 3α5α20α-hexahydroprogesterone; 3β5α20α-HHP, 3β5α20α-hexahydroprogesterone; 5α20α-THP, 5α20α-tetrahydroprogesterone; ALLOPREG, allopregnanolone; ISOPREG, isopregnanolone; THDOC, 3α,5α-tetrahydrodeoxycorticosterone; ISODOC, 3β,5α-tetrahydrodeoxycorticosterone.

mTBI may cause, among others, endocrine changes by dysregulating the stress response,^16^ activating the hypothalamo-pituitary-adrenal (HPA) axis,^17^ and influencing synthesis and metabolism of steroids and neurosteroids.^18^ Neurosteroids are endogenous steroids synthesized *de novo* in the brain, which influence neural activity ranging from neuroprotective^19–22^ to anti-inflammatory,^23–25^ and anxiolytic effects.^26–28^ Neurosteroids include progesterone and its derivatives such as 3α,5α-tetrahydroprogesterone (allopregnanolone, ALLOPREG) and 3α,5α-tetrahydrodeoxycorticosterone (THDOC), and their 3β-epimers 3β,5α-tetrahydroprogesterone (isopregnanolone, ISOPREG) and 3β,5α-tetrahydrodeoxycorticosterone (ISODOC), respectively (Fig. 1). 3α-pregnanes like ALLOPREG and THDOC are potent steroid modulators of the gamma-aminobutyric acid type-A (GABA_A_) receptors and play important roles in sedation and anxiolysis.^27,29^ On the contrary, 3β-pregnanes, such as ISOPREG and ISODOC, antagonize the effects of ALLOPREG and THDOC on the GABA_A_ receptors thereby enhancing neuronal excitability.^30–32^ Further work has shown that these 3β-pregnanes can also directly and noncompetitively inhibit GABA_A_ receptors.^33^

Neurosteroids may be altered in the brain or cerebrospinal fluid in chronic neurological conditions such as in experimental models of Alzheimer’s pathology^34^ and Parkinson’s disease^35^; or following acute events such as stroke.^36^ In experimental TBI, studies have directly investigated acute alterations in progesterone and other neurosteroids using severe, focal TBI models with region-specific lesions, involving the entopallium in finches,^37^ prefrontal cortex in rats,^18^ and orbitofrontal and perirhinal cortices in female and male mice^38,39^; however, more studies on mTBI models are needed. The therapeutic potential of neurosteroids after TBI has been previously known, culminating in successful phase II clinical trials using progesterone.^40–42^ However, the failure of two phase III clinical trials^43^ suggest that more knowledge is required regarding neurosteroids, for instance, their endogenous activity as potential diagnostic biomarkers in mTBI.

Based on these existing data, we hypothesized that mTBI would lead to acute changes in local neurosteroid levels in the brain and that these changes would also be reflected in plasma. To test this hypothesis, we utilized highly sensitive gas chromatography coupled to tandem mass spectrometry (GC-MS/MS) to evaluate steroid and neurosteroid levels in the brain and plasma of male mice after experimental mTBI.

## Materials and Methods

### Animals

Thirty-three male C57Bl/6 mice aged 7–8 weeks were used for this study. They were housed in the animal facility with a 12-h light–dark cycle and controlled temperature of 20–23°C. Food and water were provided *ad libitum* in a pathogen-free unit, as defined by the criteria of the Federation of European Laboratory Animal Science Associations. Ethical approval for animal experimentation was obtained from the Government of Upper Bavaria. All animal experiments are reported in line with the ARRIVE 2.0. guidelines.

### Experimental groups

**FIG. 2.**
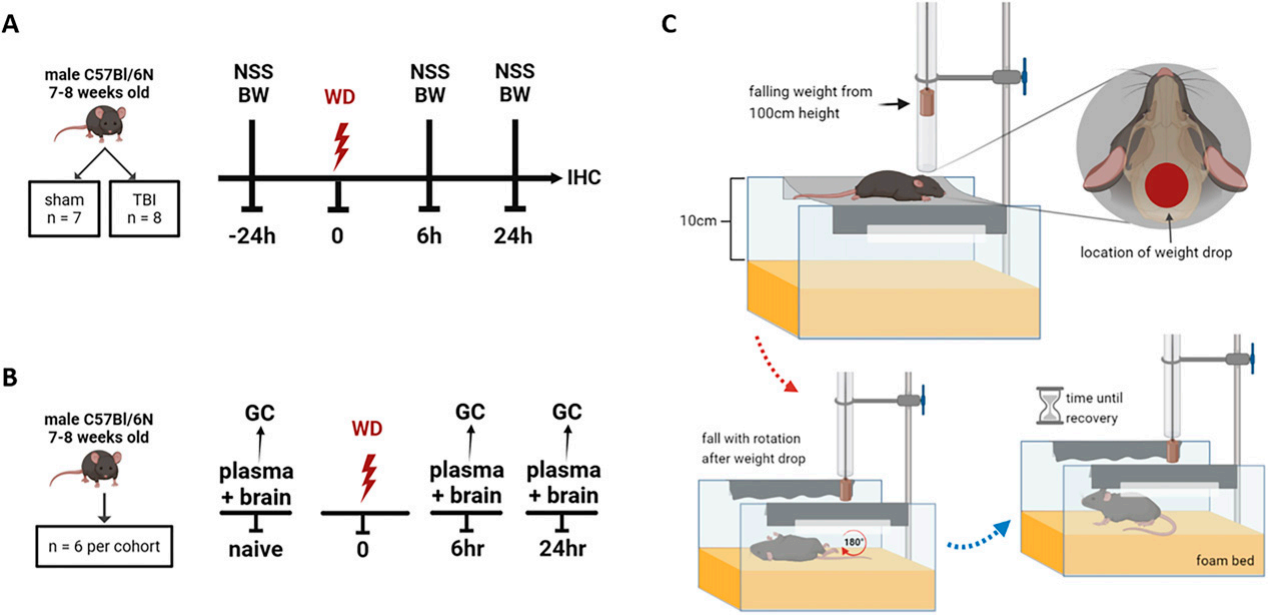
Schematic overview of the experimental protocol and weight drop model of mild traumatic brain injury (mTBI). **(A)** Experimental timeline for the neurobehavioral cohort (sham *n* = 7; TBI *n* = 8). **(B)** Experimental timeline for the neurosteroid cohort (*n* = 6 per group). **(C)** The weight drop model of mTBI, showing rotational acceleration following impact and time until recovery. NSS, neurological severity score; BW, beam walk; WD, weight drop; IHC, immunohistochemistry; GC, gas chromatography-tandem mass spectrometry.

The study included two experimental cohorts—the neurobehavioral (Fig. 2A) and the neurosteroid cohort (Fig. 2B). For the neurobehavioral cohort, the mice were randomized into sham (*n* = 7) and mTBI groups (*n* = 8) and longitudinal testing included the neurological severity score (NSS) and beam-walk testing. The neurosteroid cohort were not subjected to any behavioral testing to minimize stress—they were randomized into three groups: naive, 6 h, and 24 h post-mTBI (*n* = 6 each).

### mTBI model

We used a variant of the weight drop model described by Kane et al.^44^ No surgical incision or craniotomy was performed. To deliver the impact, a steel cylinder (90 g, 15 mm tip diameter) was dropped through a guide tube onto the intact mouse head, between lambda and bregma (Fig. 2C). The stage was constructed by two transparent plastic walls 10 cm above a foam bed with a thin laboratory wipe taped across them (Kimtech wipes; 20.5 × 20 cm). The mouse was briefly anesthetized (1.5% isoflurane in air for 70 sec) and placed prone on the stage directly under the tube. The weight was dropped from a height of 100 cm and a second hit was prevented by restraining the weight with a strong fishing line. On impact, the mouse broke the wipe, flipped 180 degrees in the anteroposterior axis, and landed on its back on the foam bed 10 cm below. For the sham group, the same procedures were performed without dropping the weight on the head, and the wipe was manually torn, thus flipping the mouse onto the foam bed. Immediately following weight drop, the time until mice recovered from anesthesia was recorded. A mouse was judged to have recovered when it became conscious, righted itself, and began locomotion. The same applied for sham mice that fell from the stage onto the foam bed. Apnea and irregular breathing were also assessed visually.

### Neurological severity score

The NSS was used to assess motor coordination and balance, as well as physiological behavior and alertness. It consisted of 10 subtests: (a) time to exit a circle of 30 cm diameter, (b) seeking behavior, (c) straight walk, (d) paresis, (e) startle reflex, (f) 3 cm beam walk, (g) 2 cm beam walk, (h) 1 cm beam walk, (i) beam balance, and (j) round stick balance. For every failed task, a score of 1 was assigned, therefore the higher the total score, the greater the deficit. NSS testing was performed at baseline, then at 6 and 24 h.

### Beam walk

The beam walk test for assessing motor function was performed as previously described.^45,46^ A mouse was placed at one end of a beam (1 m long and 1 cm wide) elevated 50 cm above the ground and allowed to walk to the other end. The time to traverse the beam and number of missteps were measured at baseline, then at 6 and 24 h after mTBI.

### Magnetic resonance imaging

Structural brain damage was assessed using a 3T Nanoscan PET-MRI scanner (Mediso Medical Imaging Systems, Budapest, Hungary) 24 h after mTBI as previously described.^45^ The following sequences were obtained: coronal T2-weighted imaging (2D fast-spin echo, repetition time [TR]/echo time [TE] = 10911/66.3 ms, averages 14, resolution 117 × 117 × 800 μm^3^) and diffusion-weighted imaging (2D spin echo, 30 directions with phase reversal, TR/TE = 3000/55.3 ms, averages 4, resolution 350 × 350 × 800 μm^3^).

### Immunohistochemistry

Following behavioral testing at 24 h after mTBI, animals were perfused transcardially with 4% paraformaldehyde and the brains were harvested for immunohistochemical analysis as previously described.^47^ Immunohistochemical staining for astrogliosis and microglial reactivity was carried out on free-floating 50 µm coronal sections, which were blocked and incubated in blocking buffer (0.2% bovine serum albumin, 0.02% fish gelatin from cold water fish skin, 0.3% Triton X-100 in 0.01 M phosphate-buffered saline [PBS], pH 7.2–7.4) with the following primary antibodies: Iba-1 (rabbit, Fujifilm Wako, #019–19741, 1:200), glial fibrillary acidic protein (GFAP)-Cy3 (mouse, Sigma-Aldrich #2905, 1:200), and NeuN (rabbit, Abcam, #177487, 1:100). After overnight incubation at 4°C, the sections were washed in PBS and incubated with the following secondary antibodies in buffer: donkey antirabbit coupled to Alexa-Fluor 488 (Jackson, #711546152, 1:500), and donkey antirabbit coupled to Alexa-Fluor 594 (Jackson, #711586152, 1:500). Nuclei were stained with 4′,6-diamidino-2-phenylindole (Invitrogen, #D1306, 1:10,000) in 0.01 M PBS.

Tissue sections were imaged using confocal microscopy (ZEISS LSM 880, Carl Zeiss Microscopy GmbH) with 10× and 40× magnification (depth of 8 bit). Image analysis was performed using Image J. Regions of interest include the cortex, hippocampus, corpus callosum, and striatum.

### Plasma and brain collection for steroid/neurosteroid analysis

Mice were gently handled to minimize stress. After brief isoflurane inhalation, the submandibular vein was punctured and blood was quickly sampled in heparinized Eppendorf tubes and centrifuged at 3000 × *g* for 10 min at 4°C to collect plasma. After blood sampling the mouse was immediately decapitated, the brain was quickly dissected, and the left hemisphere was weighed and frozen on dry ice. Both plasma and brain samples were stored at −20°C until neurosteroid analysis by GC-MS/MS.

### Steroid/neurosteroid measurements by GC-MS/MS

Neurosteroids were first extracted from plasma and brain tissue with methanol. To measure the neurosteroid levels, the following internal standards were added to the extracts: 2 ng of ^13^C_5_-5α-DHP for the analysis of 5α-dihdroprogesterone (5α-DHP); 2 ng of 19 nor-progesterone for the analysis of 5α-dihydrodeoxycorticosterone (5α-DHDOC); 2 ng of ^13^C_3_-progesterone for the analysis of progesterone; 5 ng of ^13^C_3_-deoxycorticosterone (DOC) for the analysis of DOC; 2 ng of ^13^C_5_-20α-DHP for the analysis of 20α-dihdroprogesterone (20α-DHP); 2 ng of ^13^C_3_-testosterone for the analysis of testosterone; 1 ng of ^5^H_2_-17β-estradiol for the analysis of 17β-estradiol; 1 ng of ^13^C_3_-androstenedione for the analysis of androstenedione (ADIONE); 2 ng of epietiocholanolone for the analysis of dehydroepiandrosterone (DHEA), 5α-dihydrotestosterone (5α-DHT), 3α/β5α-tetrahydrotestosterone (3α/β5α-THT), pregnenolone, ALLOPREG, ISOPREG, 5α20α-tetrahydroprogesterone (5α20α-THP), 3α/β5α20α-hexahydroprogesterone (3α/β5α20α-HHP), THDOC, and ISODOC; and 10 ng of ^2^H_8_-corticosterone for the analysis of corticosterone and 5α-dihydrocorticosterone (5α-DHC). Samples were purified and fractionated by solid-phase extraction with the recycling procedure, as described previously.^48^ Two fractions were collected from the high-performance liquid chromatography (HPLC) system: 5α-dihydroprogesterone (5α-DHPROG) was eluted in the first HPLC fraction (3–14 min) and was silylated with 50 µL of a mixture N-methyl-N-trimethylsilyltrifluoroacetamide/ammonium iodide/dithioerythritol (1000:2:5 vol/w/w) for 15 min at 70°C. The second fraction (14–25 min) containing all other steroids/neurosteroids was derivatized with 25 µL of heptafluorobutyric anhydride and 25 µL of anhydrous acetone for 1 h at 20°C. All fractions were dried under a stream of N2 and resuspended in heptane. The GC-MS/MS analysis was then performed using an AI 1310 autosampler, a Trace 1310 GC, and a TSQ 8000 MS/MS (Thermo Fisher Scientific) using argon as the collision gas. The GC-MS/MS analytical procedure was validated in brain and plasma of male mice, as previously described.^36^

### Statistical analysis

Data are presented as means ± standard error of the mean (SEM) and *n* depicts the number of animals per group. Statistical analysis was performed using GraphPad Prism 9 software. Using the Shapiro–Wilk test, we determined that all data were normally distributed. Wake-up time after mTBI was analyzed by unpaired *t* test while NSS and beam walk time were analyzed using two-way analysis of variance (ANOVA, with time as factor). Post-hoc Bonferroni correction for multiple comparisons was also performed. Brain and plasma neurosteroid measurements were analyzed using one-way ANOVA. A *p* value of < 0.05 was regarded to indicate a statistically significant difference between groups.

## Results

### General well-being after mTBI

**FIG. 3.**
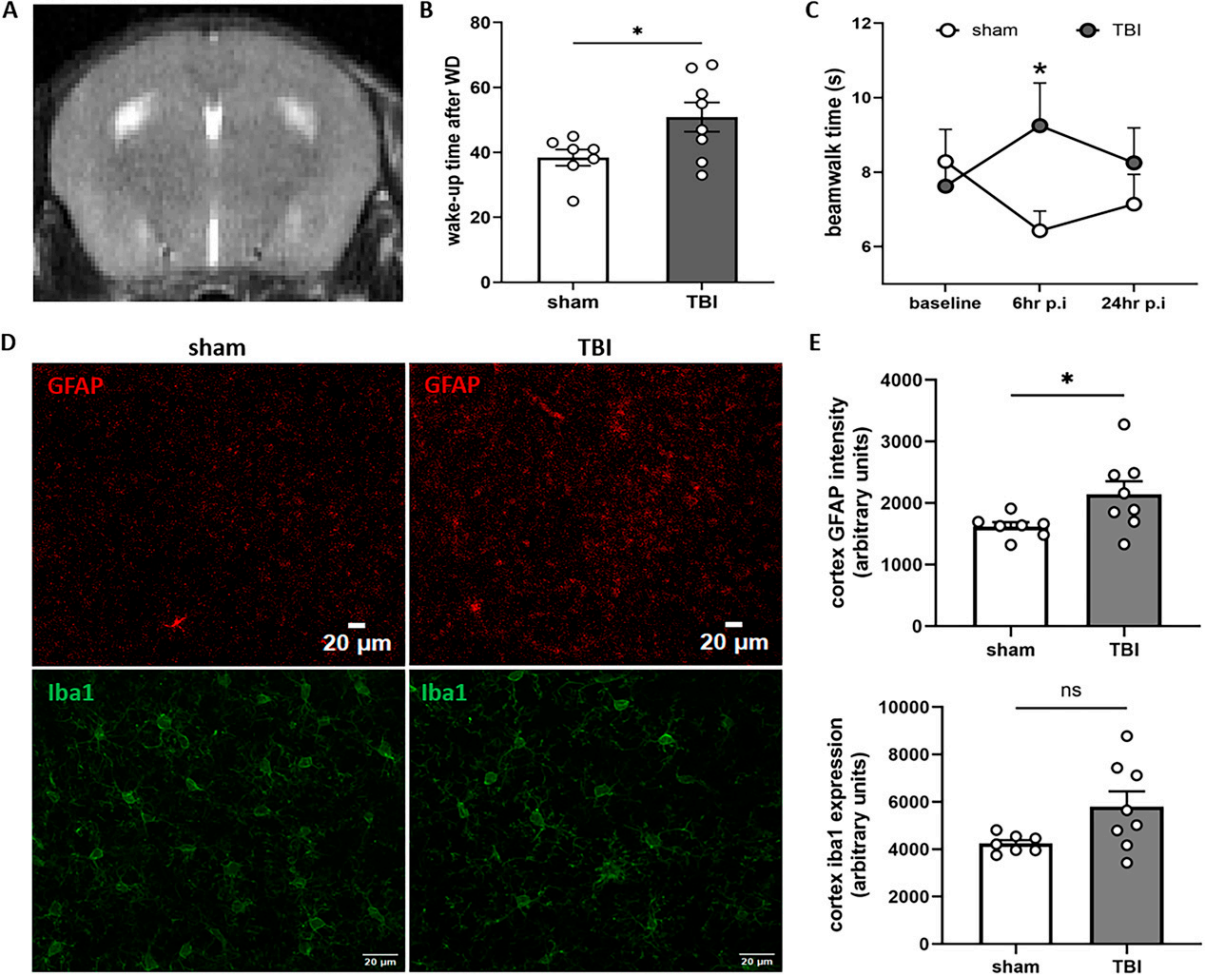
Gross pathology, wake up time, and neurological evaluation after mild traumatic brain injury (mTBI). Weight drop caused no fractures or bleeding **(A)** when observed under a dissecting microscope and by small animal magnetic resonance imaging (MRI). **(B)** Weight drop significantly increased wake up time when compared with the control (*p* < 0.05; sham *n* = 7; TBI *n* = 8). **(C)** Beam walk testing showed that mTBI significantly increased the time to cross a 1 m beam at 6 h post-mTBI (*p* < 0.05; sham *n* = 7; TBI *n* = 8), but not at 24 h. (**D, E)** Astrogliosis was increased in the cortex of mTBI mice at 24 h when compared with sham (*p* < 0.05; sham *n* = 7; TBI *n* = 8). Activated microglia were not significantly different between groups. Mean ± SEM, *p < 0.05 vs. sham.

There was no mortality following mTBI. After impact, we confirmed by visual inspection under a dissecting microscope and with animal magnetic resonance imaging that the weight drop did not cause fractures (Supplementary Fig. S1A), edema, or intracranial hemorrhage in any of the mice (Fig. 3A).

### Wake up time is significantly increased after mTBI

Following mTBI or sham injury, the mice remained on their backs until they recovered spontaneously from anesthesia and could move again. However, mice subjected to mTBI had a significantly increased wake up time when compared with their sham counterparts (Fig. 3B; *p* < 0.05).

### mTBI caused transient motor deficits but not anxiety, coordination, or balance deficits

According to the NSS, animals with mTBI did not demonstrate significant deficits in anxiety, coordination, or balance when compared with their sham counterparts (Supplementary Fig. S1B). Beam walk testing, however, showed that the time needed to cross the beam was significantly increased 6 h post-injury compared with the control (*p* < 0.05). No significant change was observed at 24 h (Fig. 3C). No significant changes were observed in the number of foot slips (Supplementary Fig. S1C).

### mTBI leads to acute astrogliosis but not activation of microglia

The intensity of GFAP staining was increased in the cortex of mTBI mice when compared with the sham group (Fig. 3D and E, upper row; *p* < 0.05). GFAP staining in the corpus callosum and hippocampus was not different between groups (data not shown). Intensity for the microglial protein iba1 was not changed in the cortex (Fig. 3D and E, bottom row), hippocampus, or striatum (data not shown).

### mTBI caused significant changes in brain neurosteroid levels

**FIG. 4.**
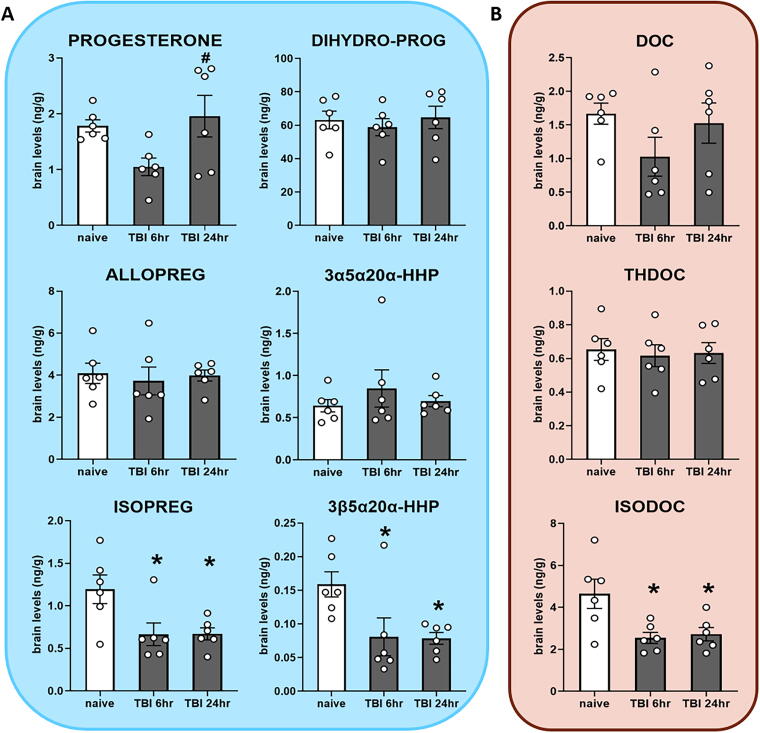
Steroid and neurosteroid levels in brain after mild traumatic brain injury (mTBI), measured by gas chromatography-tandem mass spectrometry (GC-MS/MS) in naive mice (*n* = 6), mTBI mice at 6 h (*n* = 6), and 24 h (*n* = 6). **(A)** Concentrations of progesterone and reduced metabolites. **(B)** Concentrations of deoxycorticosterone (DOC) and reduced metabolites. 3α5α20α-HHP, 3α5α20α-hexahydroprogesterone; 3β5α20α-HHP, 3β5α20α-hexahydroprogesterone. Mean ± SEM. *p < 0.05 vs. naive; ^#^*p* < 0.05 vs. TBI 6 h.

All measured neurosteroid levels in the brain are shown in Table 1. Progesterone was not significantly different when compared with the control (Fig. 4A, top row). Whereas ALLOPREG and 3α5α20α-HHP showed no significant differences between groups, their 3β-counterparts ISOPREG and 3β5α20α-HHP showed significantly reduced levels for the 6 and 24 h groups when compared with naive (Fig. 4a, bottom row; *p* < 0.05). The neurosteroid THDOC showed no significant difference after mTBI while its 3β-epimer ISODOC was significantly reduced in the 6 and 24 h groups compared with the naive (Fig. 4B, bottom row; *p* < 0.05).

**Table 1.** Steroid and Neurosteroid Levels in Brain After mTBI

Neurosteroid	Naive	TBI 6 h	TBI 24 h	*p* value
Pregnenolone	7.45 ± 0.82	5.70 ± 0.73	7.74 ± 0.82	0.037*
Progesterone	1.78 ± 0.11	1.05 ± 0.16	1.96 ± 0.37	0.041*
5α-DHP	63.18 ± 5.30	58.84 ± 5.12	64.69 ± 6.72	0.760
ALLOPREG	4.09 ± 0.48	3.99 ± 0.26	3.73 ± 0.66	0.871
ISOPREG	1.20 ± 0.17	0.67 ± 0.07	0.67 ± 0.13	0.017*
20α-DHP	0.59 ± 0.09	0.68 ± 0.16	0.25 ± 0.06	0.041
3α5α20α-HHP	0.64 ± 0.07	0.69 ± 0.07	0.85 ± 0.22	0.573
3β5α20α-HHP	0.16 ± 0.02	0.08 ± 0.01	0.08 ± 0.03	0.020*
5α20α-THP	0.94 ± 0.10	0.90 ± 0.07	0.98 ± 0.13	0.860
DOC	1.67 ± 0.16	1.53 ± 0.30	1.03 ± 0.29	0.213
THDOC	0.65 ± 0.07	0.63 ± 0.06	0.62 ± 0.07	0.919
ISODOC	4.65 ± 0.70	2.73 ± 0.32	2.55 ± 0.26	0.011*
Corticosterone	79.37 ± 7.99	102.3 ± 17.67	66.28 ± 9.89	0.157
5α-DHC	3.41 ± 0.35	6.80 ± 0.49	7.46 ± 1.08	0.002**
DHEA	0.09 ± 0.01	0.08 ± 0.01	0.07 ± 0.01	0.263
Adione	0.13 ± 0.08	0.11 ± 0.01	0.12 ± 0.06	0.985
Testosterone	0.59 ± 0.44	0.59 ± 0.50	1.08 ± 0.58	0.732
3α5α-THT	0.14 ± 0.09	0.12 ± 0.09	0.18 ± 0.08	0.888
3β5α-THT	0.04 ± 0.03	0.02 ± 0.02	0.05 ± 0.02	0.647
Estradiol	0.005 ± 0.002	0.005 ± 0.001	0.007 ± 0.003	0.871

mTBI, mild traumatic brain injury; 5α-DHP, 5α-dihydroprogesterone; ALLOPREG, allopregnanolone; ISOPREG, isopregnanolone; 20α-DHP, 20α-dihydroprogesterone; 3α5α20α-HHP, 3α5α20α-hexahydroprogesterone; 3β5α20α-HHP, 3β5α20α-hexahydroprogesterone; 5α20α-THP, 5α20α-tetrahydroprogesterone; DOC, deoxycorticosterone; THDOC, 3α5α-tetrahydrodeoxycorticosterone; ISODOC, 3β5α-tetrahydrodeoxycorticosterone; 5α-DHC, 5α-dihydrocorticosterone; DHEA, dehydroepiandrosterone; Adione, androstenedione; 3α5α-THT, 3α5α-tetrahydrotestosterone; 3β5α-THT, 3β5α-tetrahydrotestosterone. *p < 0.05, **p < 0.01.

### mTBI caused significant changes in plasma neurosteroid levels

**FIG. 5.**
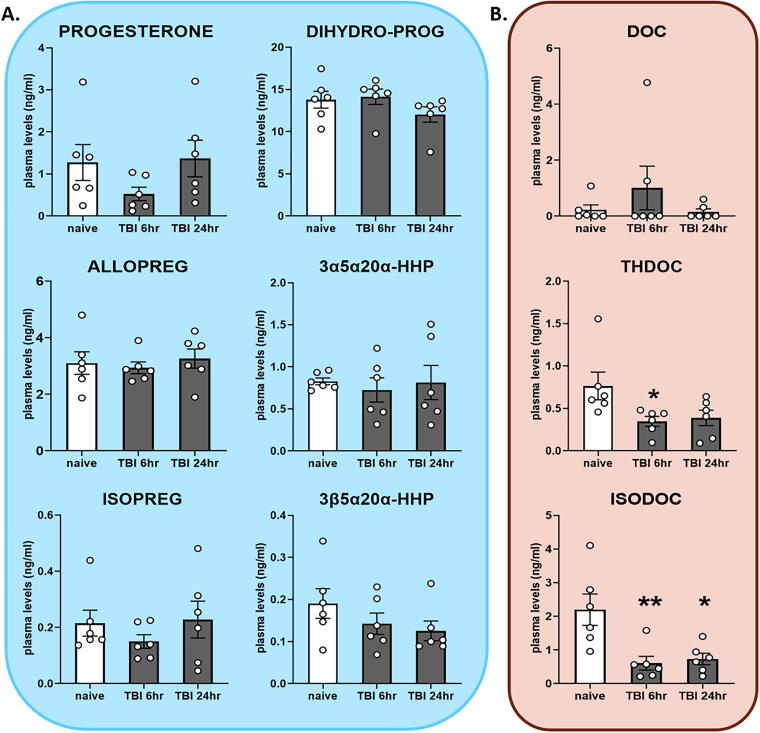
Steroid and neurosteroid levels in plasma after mild traumatic brain injury (mTBI), measured by gas chromatography-tandem mass spectrometry (GC-MS/MS) in naive mice (*n* = 6), mTBI mice at 6 h (*n* = 6), and 24 h (*n* = 6). **(A)** Concentrations of progesterone and reduced metabolites. **(B)** Concentrations of deoxycorticosterone (DOC) and reduced metabolites. 3α5α20α-HHP, 3α5α20α-hexahydroprogesterone; 3β5α20α-HHP, 3β5α20α-hexahydroprogesterone. Mean ± SEM. *p < 0.05 vs. naive; **p < 0.01 vs. naive.

All measured neurosteroid levels in plasma are shown in Table 2. Progesterone, ALLOPREG, and ISOPREG were not significantly altered after mTBI (Fig. 5A). THDOC was significantly reduced in the 6 h group (Fig. 5B, middle row; *p* < 0.05), while ISODOC was significantly reduced in both the 6 and 24 h groups compared with the control (Fig. 5B, bottom row; *p* < 0.01 and *p* < 0.05).

**Table 2.** Steroid and Neurosteroid Levels in Plasma After mTBI

Neurosteroid	Naive	TBI 6 h	TBI 24 h	*p* value
Pregnenolone	1.20 ± 0.16	0.66 ± 0.12	1.03 ± 0.21	0.103
Progesterone	1.27 ± 0.43	0.53 ± 0.16	1.37 ± 0.44	0.234
5α-DHP	13.79 ± 1.00	14.14 ± 0.91	12.04 ± 0.91	0.260
ALLOPREG	3.10 ± 0.40	2.94 ± 0.21	3.26 ± 0.34	0.785
ISOPREG	0.22 ± 0.05	0.15 ± 0.03	0.23 ± 0.07	0.490
20α-DHP	0.33 ± 0.01	0.24 ± 0.06	0.23 ± 0.144	0.766
3α5α20α-HHP	0.83 ± 0.04	0.73 ± 0.14	0.81 ± 0.203	0.866
3β5α20α-HHP	0.19 ± 0.04	0.14 ± 0.03	0.13 ± 0.023	0.278
5α20α-THP	0.56 ± 0.20	0.49 ± 0.12	0.80 ± 0.215	0.468
DOC	0.23 ± 0.17	1.01 ± 0.78	0.16 ± 0.103	0.380
THDOC	0.76 ± 0.16	0.35 ± 0.06	0.39 ± 0.091	0.038*
ISODOC	2.10 ± 0.47	0.61 ± 0.21	0.73 ± 0.171	0.004**
Corticosterone	116.50 ± 15.12	245.90 ± 80.59	262.50 ± 70.79	0.228
5α-DHC	4.45 ± 0.99	20.10 ± 8.47	15.74 ± 3.53	0.135
DHEA	0.01 ± 0.05	0.06 ± 0.01	0.07 ± 0.014	0.634
Adione	0.18 ± 0.14	0.17 ± 0.09	0.14 ± 0.043	0.947
Testosterone	0.94 ± 0.77	1.32 ± 0.70	0.68 ± 0.608	0.810
3α5α-THT	0.15 ± 0.09	0.27 ± 0.08	0.11 ± 0.065	0.404
3β5α-THT	0.19 ± 0.11	0.24 ± 0.09	0.11 ± 0.077	0.632
estradiol	0.020 ± 0.010	0.030 ± 0.010	0.003 ± 0.001	0.368

mTBI, mild traumatic brain injury; 5α-DHP, 5α-dihydroprogesterone; ALLOPREG, allopregnanolone; ISOPREG, isopregnanolone; 20α-DHP, 20α-dihydroprogesterone; 3α5α20α-HHP, 3α5α20α-hexahydroprogesterone; 3β5α20α-HHP, 3β5α20α-hexahydroprogesterone; 5α20α-THP, 5α20α-tetrahydroprogesterone; DOC, deoxycorticosterone; THDOC, 3α5α-tetrahydrodeoxycorticosterone; ISODOC, 3β5α-tetrahydrodeoxycorticosterone; 5α-DHC, 5α-dihydrocorticosterone; DHEA, dehydroepiandrosterone; Adione, androstenedione; 3α5α-THT, 3α5α-tetrahydrotestosterone; 3β5α-THT, 3β5α-tetrahydrotestosterone. *p < 0.05, **p < 0.01.

### Neurosteroids more abundant in brain than in plasma

**FIG. 6.**
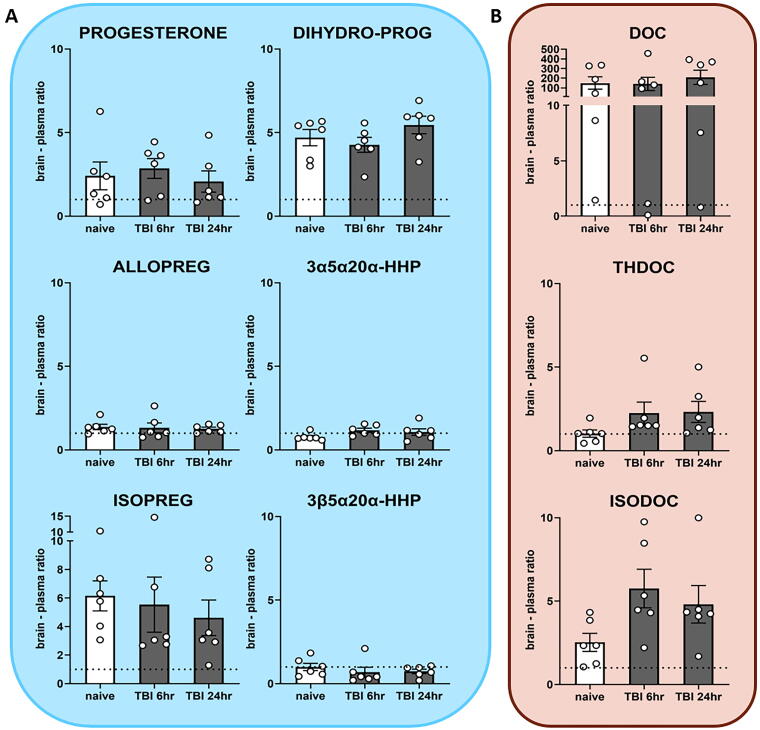
Brain–plasma ratio of neurosteroids after mild traumatic brain injury (mTBI). Dotted line signifies equal brain–plasma ratio. DOC, deoxycorticosterone; 3α5α20α-HHP, 3α5α20α-hexahydroprogesterone; 3β5α20α-HHP, 3β5α20α-hexahydroprogesterone. Mean ± SEM.

We calculated the brain–plasma ratio to evaluate the relative abundance of neurosteroids in the brain. Values greater than 1 indicated more abundance in brain while values less than 1 indicated more abundance in plasma (Fig. 6). Progesterone and its reduced metabolites were more abundant in the brain than plasma, except for ALLOPREG, 3α5α20α-HHP, and 3β5α20α-HHP, which had approximately equal concentrations in brain and plasma (Fig. 6A). The DOC derivatives, on the contrary, had equal brain–plasma ratios, apart from DOC and THDOC, which were more abundant in the brain than in plasma (Fig. 6B). Taken together, the majority of neurosteroids were found in greater or equal ratios in brain than in plasma.

## Discussion

Our study investigated the impact of mTBI on neurosteroid levels in the brain and plasma of male mice. We observed that mTBI led to significantly reduced neurosteroid levels in brain and plasma, even in the absence of gross brain pathology. More specifically, we noted that the concentration of 3β-pregnanes such as ISOPREG, 3β5α20α-HHP, and ISODOC were reduced in brain while THDOC and ISODOC levels were reduced in plasma. Because these neurosteroids (except THDOC) are catalyzed by the same 3β-hydroxysteroid oxidoreductase (3β-HSOR) enzyme,^18,49^ and because they also modulate GABA_A_ receptors (except 3β5α20α-HHP),^30,31^ our results do not only identify ISODOC to be a potential diagnostic blood biomarker for mTBI but also highlight a possible mechanism of how neurosteroids may be involved in the pathophysiology of mTBI.

Our study utilized a murine model of weight drop that combines impact with rotational acceleration. Animal models of TBI are important for delineating various aspects of the injury cascade, which may be difficult or nearly impossible to examine in a patient population.^50^ We reported physiological or functional alterations after mTBI but no gross pathology such as skull fracture, edema, or hemorrhage. We observed astrogliosis in the cortex, the brain region located directly underneath the impact site, 24 h after mTBI. Areas further away from the impact site showed no gliosis. Microglial reactivity was not changed in any of the investigated brain regions. These data suggest that the activation of astrocytes after mTBI may precede microglial activation and may thus be the first agent of neuroinflammation. Chronic TBI studies suggest that microglial activation persist while astrogliosis recedes. Our weight drop model is clinically relevant as it mirrors a population of patients with normal structural neuroimaging after mTBI, which may still experience acute symptoms and are at risk for developing chronic symptoms later on.^51,52^

When compared with control, mice with mTBI showed an increase in wake-up time from anesthesia, which suggests loss of consciousness during anesthesia. Whereas loss of consciousness is not a strict requirement for the diagnosis of mTBI in humans, it is nevertheless important in our study as it confirms immediately that an mTBI has indeed taken place. The wake-up time is not to be confused with spontaneous righting reflex since wake-up time also incorporates motor behavior. Seeing that spontaneous recovery may occur without cortical activity, the wake-up time involving motor behavior is a more robust measure of arousal.^53^ NSS testing showed no anxiety or balance deficits in the injured group when compared with the control while 1 m beam walk testing showed a transient delay in crossing the beam after mTBI. This confirms that loss of consciousness may occur following mTBI with little or no accompanying acute neuropathology.^38,44,54,55^ Although our findings may seem to corroborate reports regarding mTBI mostly as a disorder of function rather than structure,^5,56^ advanced neuroimaging such as diffusion tensor and kurtosis imaging have already challenged this view.^50,57,58^ mTBI may thus be more accurately assessed as a disorder including cognitive as well as microstructural alterations, rather than an isolated paradigm.

Following mTBI, there was a significant reduction in brain levels of ISOPREG, 3β,5α,20α-HHP, and ISODOC at 6 and 24 h. Since they are all catalyzed by the same enzyme, this finding suggests that mTBI may result in the downregulation of one specific enzyme. An earlier study showed that an isotype of 3β-HSOR, 3β-hydroxysteroid dehydrogenase, had significantly reduced mRNA expression in rat brain after bilateral contusion TBI.^59^ Furthermore, ISOPREG and ISODOC are implicated in neuronal (hyper-)excitability by directly antagonizing GABA_A_ receptors,^33^ which seems highly likely in our study since ALLOPREG and THDOC levels remain unchanged. We thus propose that enzymatic downregulation of 3β-HSOR and direct disinhibition of GABAergic neuronal inhibition may be possible mechanisms of mTBI, or a potential cerebroprotective mechanism by a GABAergic response upregulation. Our rationale is in line with a study that reported a significant reduction in ISOPREG levels after TBI in female mice.^38^ Reduction of brain ISOPREG levels, however, do not seem to be a general indicator of brain injury since ISOPREG levels increased in the cerebral cortex of mice after experimental stroke^36^ and chronic experimental autoimmune encephalomyelitis.^60^ Hence, reduction of brain ISOPREG levels seem to be rather specific for mTBI.

In plasma, however, THDOC is reduced 6 h after mTBI and ISODOC is reduced both at 6 and 24 h after mTBI. We suggest that ISODOC acts as a negative allosteric modulator of the GABA_A_ receptor because THDOC is simultaneously affected. Reduction of ISODOC levels even at 24 h may stimulate the action of the GABA_A_ receptor leading to increased neuronal GABAergic inhibition. Our result is in contrast to a study that showed a threefold increase in plasma THDOC levels during stress,^61^ thus reinforcing the idea that mTBI may influence neurosteroid response quite differently from stroke or stress. Since acute stress may increase neurosteroid levels while chronic stress leads to significant downregulation, our data suggest a peculiar mechanism of mTBI with respect to neurosteroid levels.

Finally, we calculated the brain–plasma neurosteroid ratio after mTBI. We observed that most pregnane neurosteroids including ISOPREG and ISODOC were more abundant in the brain than plasma, signifying a major local neurosteroid activity in the brain and minimal influence from systemic circulation.^18^ For ALLOPREG and 3β5α20α-HHP, the brain levels were equal with plasma levels, suggesting possible systemic influence, especially for the former, which is a key player in the HPA axis response.^62,63^

Being able to measure neurosteroid changes in plasma is critical for possible biomarker studies, thus the optimal neurosteroid biomarker should be easily measurable in plasma as well as mirror the concentration in the brain. In our study, reduced ISODOC levels in brain after mTBI are simultaneously reflected in the plasma at both 6 and 24 h. Because ISODOC appears more abundantly in the brain than in plasma, it is most likely of cerebral origin. Further evaluation is needed in order to effectively characterize ISODOC as a potential diagnostic mTBI biomarker.

Limitations of the study include use of male mice only, lack of longitudinal neurosteroid measurements, and use of entire cerebral hemispheres for analysis. First, we used only male mice to keep away the confounding factors of hormonal cycle changes in females, thus requiring further studies including female mice. Second, we did not perform longitudinal neurosteroid measurements using the same animals because we had to harvest the brains simultaneously. This afforded us the opportunity to compare local neurosteroid activity with systemic neurosteroid response. After this screening process, we plan simultaneous longitudinal measurements where only the plasma would be collected intermittently. A final limitation was that we used the entire hemispheres for neurosteroid analysis, which did not allow us to investigate region-specific differences in brain neurosteroid levels. The high sensitivity inherent to the GC-MS/MS technology, down to the attomole, will permit us to investigate specific brain structures in the future.

## Conclusions

Our study showed that the neurosteroids ISOPREG and ISODOC, which are both synthetized by 3β-HSOR, are significantly reduced in the brain acutely after experimental mTBI. In plasma, ISODOC levels are reduced also. Hence, we identified the enzyme 3β-HSOR to be specifically downregulated by mTBI and its product, ISODOC, as a potential diagnostic plasma biomarker for mTBI.

### Transparency, Rigor, and Reproducibility Statement

All experiments in our study adhered to the protocols approved by the Government of Upper Bavaria. This included the choice of species and sex, randomization and blinding, and group size calculation. Accordingly, 33 male wild-type C57Bl/6N mice were used in our study—15 mice were randomized into sham (*n* = 7) and mTBI (*n* = 8) for neurobehavioral testing (NSS and 1 m beam walk) while 18 mice were randomized into naive, 6 h-mTBI, and 24 h-mTBI groups for neurosteroid analysis (*n* = 6 per group). Randomization was done using an online randomizing tool (https://www.randomizer.org). mTBI was administered by dropping a steel cylinder of 90 g on the head of a briefly anesthetized mouse (5% isoflurane in 1 L/min room air for 70 sec) and time until wake up from anesthesia was assessed. Afterward, the mice were sacrificed and brains and plasma collected for neurosteroid analysis using the highly specific gas chromatography (Trace 1310 GC) coupled to tandem mass spectrometer (TSQ 8000 MS/MS). All brain and plasma samples were obtained by the experimenter. We plan further experiments using female mice. This article will be published under a Creative Commons Open Access license and will be freely available upon publication.

## Abbreviations Used

HPLC: high-performance liquid chromatography
mTBI: mild traumatic brain injury
SEM: standard error of the mean
GFAP: glial fibrillary acidic protein
