# Supplementary material for: Mild TBI Changes Brain and Plasma Neurosteroid Levels in Mice
Source: Neurotrauma Rep. 2025 Jan 20;6(1):39–52. doi: 10.1089/neur.2024.0151 (PMC11839540; doi:10.1089/neur.2024.0151)
Supplement: Supplementary Figure S1 [file neur.2024.0151_supp_figs1.docx]

**
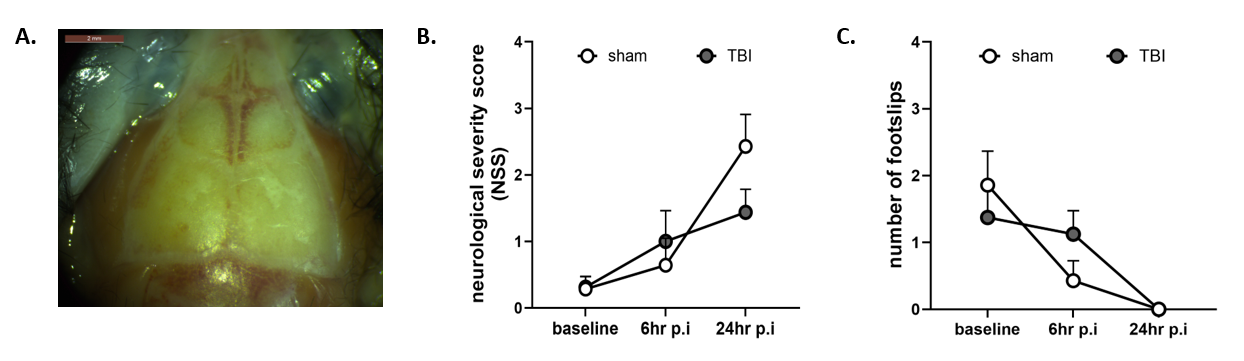
**

**Suppl. Figure 1:** Gross pathology after mTBI. **A.** There were no fractures after mTBI as assessed by visual inspection. **B.** Neurological severity score (NSS) testing showed no behavioral deficits after mTBI when compared to the control (sham n = 7; TBI n = 8). **C.** There was no significant difference in number of foot slips on the 1m beam between groups (sham n = 7; TBI n = 8).. Mean ± SEM
